# Notes and News

**Published:** 1902-05-31

**Authors:** 


### Notes and News.

The sudden rise of temperature in London on Saturday brought a good many patients to the hospitals suffering directly from the effects of heat.

Small-pox still lingers in Swansea, and recently difficulties have been placed in the way of the officials removing patients to the hospital.

Surgeon-Captain T. J. Crean, V.C., has been made an Honorary Fellow of the Royal College of Surgeons in Ireland.

A Committee on Ventilation has been appointed for the Houses of Parliament. From statements made before Parliament recently it appears to be very necessary that the general ventilation and cleaning of the chambers should be under one authority, which has not been the case hitherto.

We learn that a generous supporter has promised the Hospital Sunday Fund a gift equal to one-fourth of the amount collected in the places of worship on June 15th, so that every sovereign given in the churches and chapels will ensure an additional five shillings going to the hospitals and dispensaries.

Of the 713 fatal cases of lightning stroke reported in the United States during 1900, 291 persons were killed in the open, 158 in houses, 57 under trees, and 56 in barns. The circumstances attending the death of the remaining 151 were not reported. This seems to dispose of the old superstition that the safest place to be in during a thunderstorm is the open country and the most dangerous under a tree.

A Matinée will be given at 2 o'clock on Friday, June 13th, at the Lyric Theatre in aid of the League of Mercy. Amongst the Patronesses are the Duchess of Marlborough, the Dowager Duchess of Argyll, the Marchioness of Hastings, the Countesses of Winchilsea, of Warwick, and Countess Cadogan. The programme is a most attractive one, in which Miss Kate Rorke, Mrs. Bernard Beere, Miss Lottie Venne, Mr. Forbes Robertson, Mr. Hayden Coffin, and Mr. Albert Chevalier will appear. Miss Esther Palliser and her sister Miss May Palliser will sing. The League of Mercy secured £10,000 for the hospitals last year, and is a valuable worker for the King's Hospital Fund.

Although the small-pox epidemic in the Essex direction shows less signs of spreading, in the Metropolis generally the decrease is not marked, but the numbers show considerable variations from day to day.

The Royal Society has sent out a commission to Uganda to investigate sleeping sickness. Doctors G. C. Low and Christy will conduct the parasitological investigations and Dr. Castelani those connected with bacteriology.

At the Westminster Hospital post-graduate course the clinical demonstration on Tuesday, June 3rd, will be by Dr. W. A. Wills, on "A Case of Raynaud's Disease with Sclerodermia, with some other illustrative cases." The hour of the demonstration is 4.30 p.m.

A double number of The Hospital will be issued on June 14th, as the Special Coronation Hospital Sunday Number. It will be accompanied by two beautiful photogravure plates, being portraits of the Prince and Princess of Wales, with autographic signatures. The price of the double number will be 4d. Subscribers desiring to receive the plates must send two penny stamps to the Manager of The Hospital, 28 Southampton Street, Strand, before June 12th.

The difficulty created at Torbay Hospital by the resignation of the Weekly Board, in consequence of the recent dispute with the medical staff, has now been practically overcome by the appointment of a body of gentlemen who are likely to have the confidence of the public. Captain Phillpotts, one of the new Board, has previously served the institution as its hon. secretary, and as the rest of the board is constituted of gentlemen of position and influence in Torquay, there is good reason to believe that, while its interests are safeguarded, their appointment will be acceptable to all.

We are asked to announce that the provincial meeting of the Society for the Study of Disease in Children will be held at the Pendlebury Children's Hospital, Manchester, this year, on Friday, June 20th, at 4 p.m. At the conclusion of the meeting the members and their friends will dine at the Queen's Hotel. The following day an excursion into Derbyshire has been so arranged that members will be able to return to town the same night, and those who intend to be present are requested to communicate with Dr. George Carpenter, Welbeck Street, W., as soon as possible.

The mortality from plague in India is becoming less severe. The Punjaub is still the most fatal district, but even here a very marked decline is shown. At Marseilles, the great port for the East, the quarantine and disinfecting regulations are continually being improved and rendered more effective. Formerly they served little other purpose than to inconvenience and annoy. Ships arriving from a plague-infested country must stop at the island of Frioul, where passengers are examined. If an actual case of plague is discovered, ten days' quarantine is imposed upon the ship. If no plague exists, and linen can be disinfected upon the ship, first-class passengers are allowed to proceed and land. Emigrants and steerage passengers have to submit to more stringent regulations.

